# Delta radiomics analysis of Magnetic Resonance guided radiotherapy imaging data can enable treatment response prediction in pancreatic cancer

**DOI:** 10.1186/s13014-021-01957-5

**Published:** 2021-12-15

**Authors:** M. R. Tomaszewski, K. Latifi, E. Boyer, R. F. Palm, I. El Naqa, E. G. Moros, S. E. Hoffe, S. A. Rosenberg, J. M. Frakes, R. J. Gillies

**Affiliations:** 1 Cancer Physiology Department, H. Lee Moffitt Cancer Center and Research Institute, 12902 USF Magnolia Dr, Tampa, FL 33612 USA; 2 Medical Physics Department, H. Lee Moffitt Cancer Center and Research Institute, Tampa, FL USA; 3 Radiation Oncology Department, H. Lee Moffitt Cancer Center and Research Institute, Tampa, FL USA; 4 Machine Learning Department, H. Lee Moffitt Cancer Center and Research Institute, Tampa, FL USA; 5 Present Address: Translation Imaging Department, Merck & Co, West Point, PA USA

## Abstract

**Background:**

Magnetic Resonance Image guided Stereotactic body radiotherapy (MRgRT) is an emerging technology that is increasingly used in treatment of visceral cancers, such as pancreatic adenocarcinoma (PDAC). Given the variable response rates and short progression times of PDAC, there is an unmet clinical need for a method to assess early RT response that may allow better prescription personalization. We hypothesize that quantitative image feature analysis (radiomics) of the longitudinal MR scans acquired before and during MRgRT may be used to extract information related to early treatment response.

**Methods:**

Histogram and texture radiomic features (n = 73) were extracted from the Gross Tumor Volume (GTV) in 0.35T MRgRT scans of 26 locally advanced and borderline resectable PDAC patients treated with 50 Gy RT in 5 fractions. Feature ratios between first (F1) and last (F5) fraction scan were correlated with progression free survival (PFS). Feature stability was assessed through region of interest (ROI) perturbation.

**Results:**

Linear normalization of image intensity to median kidney value showed improved reproducibility of feature quantification. Histogram skewness change during treatment showed significant association with PFS (*p* = 0.005, HR = 2.75), offering a potential predictive biomarker of RT response. Stability analyses revealed a wide distribution of feature sensitivities to ROI delineation and was able to identify features that were robust to variability in contouring.

**Conclusions:**

This study presents a proof-of-concept for the use of quantitative image analysis in MRgRT for treatment response prediction and providing an analysis pipeline that can be utilized in future MRgRT radiomic studies.

**Supplementary Information:**

The online version contains supplementary material available at 10.1186/s13014-021-01957-5.

## Introduction

Pancreatic cancer is a deadly disease with survival statistics remaining poor despite intensive research. Locally advanced Pancreatic Ductal Adenocarcinoma (PDAC) has a dismal 5-year survival rate of around 9% [1], with approximately 1/3 of patients expected to die of local progression [2]. Evaluating therapeutic response of local disease as early as possible is therefore crucial, as maximization of the therapeutic impact to the primary site is critical. For locally advanced patients, evidence has been accumulating that hypofractionated radiotherapy, i.e. stereotactic body radiotherapy (SBRT), can improve both local control and overall survival for these patients [3]. However, it remains a significant challenge to deliver high dose SBRT to pancreatic tumors given their motion and their close proximity to normal structures, such as bowel, that are sources of dose-limiting toxicity [4]. MRI guided radiotherapy (MRgRT) is a newer form of radiotherapy that allows for real-time adaptive treatment, tracking of tumor/normal organs, and allows dose escalation to improve the therapeutic window [5].

The excellent soft tissue contrast of MR methods compared to CT provides an opportunity for a dramatic increase in the role of MR in radiotherapy response detection. Through real time imaging, MRgRT technology supplies a dynamic imaging record of tumor changes with every radiation treatment. This information has been used purely for improved planning (better tissue segmentation) and patient setup for radiation delivery. We hypothesize that the wealth of image data provided by MRgRT could also be used to provide a quantitative assessment of intermediate responses and direct the subsequent treatment fractions accordingly. Understanding the patterns of response through automated analysis of imaging data will enable closer monitoring of the tumor changes after irradiation, informing clinical decisions. This is particularly relevant in the setting of those tumors that significantly involve the adjacent vasculature such as those patients with locally advanced pancreatic cancer (LAPC); if enough regression occurs, these tumors may become resectable with negative margins (R0 resection). Decision making at the 4–6 week time point post treatment involves multidisciplinary review which is often complicated by the post MRgRT soft tissue edema. Although the decision to proceed with resection is highly subjective as no consensus guidelines exist [6], some institutions have developed tools to improve prediction of resectablility, including data showing that 20% of LAPC patients ultimately could be resected with an improved median overall survival [7]. Automated analysis of the imaging data during treatment could increase the reliability of predicting SBRT success and impact the decision to proceed with resection. PDAC patients may benefit particularly from the advances in MR-based therapy response assessment given their high risk of failure, especially in the unresectable cases.

The emerging field of machine learning in image analytics, or “radiomics”, can provide powerful tools to enable such analysis of the dense MRgRT data. Employing automated high-throughput image feature extraction methods to quantify subtle patterns in the daily images, radiomic analyses [8] have shown great promise for disease prognosis or prediction of treatment response, including radiotherapy [9]. In early proof-of-concept MRgRT radiomic studies, Boldrini et al. [10] considered changes in extracted features using ratios pre- to post-treatment in 16 rectal cancer patients treated on a 0.35T MRgRT system showing promise for response correlation, later they followed up with another study in pancreatic cancer [11]. Simpson et al. [12] looked at static, absolute values of radiomic features derived from 0.35T MRgRT scans for response prediction in 20 pancreatic cancer patients. With the increasing interest and promise in the field, there is an unmet need for establishing a robust MRgRT image quantification framework to extract the informational content of the imaging data and ensure reproducibility and reliability of extracted features.

Unlike Computed Tomography or Positron Emission Tomography, where pixel/voxel values are related to physical and/or functional properties of the object, absolute MRI signal intensities have no inherent biophysical meaning thereby complicating their radiomic analysis. Image pre-processing and normalization are usually required for efficient quantification of MR signal intensities [13]. Development of an appropriate normalization framework is therefore crucial also for quantitative analysis of MRgRT image data, yet has not been discussed before specifically in the context of this technology. Therefore, in this study an image processing and normalization framework is developed, and feature robustness assessed, with the aim of simplifying and improving the robustness of future MRgRT radiomics analyses.

The short history of MRgRT in routine clinical use limits the availability of patient data, affecting the study design and statistical approaches used in this work. Multivariate, machine learning radiomic analyses cannot confidently be applied in such studies highlighting the importance of feature stability and validation to ensure reproducibility and minimize overfitting [14]. The work described herein is focused on quantification and univariate analysis of changes in standardized [15] radiomic (histogram and texture) image features during treatment.

In this study, we present a robust radiomic framework for quantification of image changes during radiotherapy in pancreatic adenocarcinoma, demonstrating the feasibility of the approach by successful prediction of disease progression in a cohort of 26 borderline/locally advanced PDAC patients who did not undergo surgery after SBRT.

## Methods

### Data collection

The Institutional Review Board at the University of South Florida approved (IRB #20383) and waived the informed consent requirement for retrospective analysis in this study.

Imaging and follow-up data were compiled from a total of n = 26 patients treated with 5 fractions of SBRT on the MRidian (ViewRay Inc., Cleveland, OH) magnetic resonance guided radiotherapy system (also known as a MRI-Linac system) at the Moffitt Cancer Center and that did not undergo resection. The patients were treated to a median dose of 50 Gy to Gross Tumor Volume (GTV). Progression Free Survival (PFS) was quantified time from MRgRT to latest follow-up or a (local or distant) progression event. Local progression was assessed via RECIST 1.1 using largest tumor diameter in CT scans first obtained approximately 1 month after RT and then every 3 months for one year. An increase of 20% or more represented progressive disease, and a decrease of 30% or more represented partial response. Stable disease was defined as any change between a 20% increase and a 30% decrease. Distant progression was determined by US guided biopsy confirming metastatic disease (most often to the liver), pathology confirmed nodules discovered during exploratory laparotomy (once in liver, once in peritoneum), or by enlarged nodules outside the pancreas on imaging (two instances). Patient demographics are detailed in Table 1.

**Table 1:** Patient information

Age (years)	66 (60–72)
Sex	
Female	12 (46%)
Male	14 (54%)
Histology	
Adenocarcinoma	26 (100%)
Tumor location	
Head	21 (81%)
Body	4 (15%)
Tail	1 (4%)
Resectability status (at diagnosis)	
Borderline resectable	11 (42%)
Locally advanced	15 (58%)
Induction chemotherapy	
FOLFRINOX	16 (62%)
Gem/Abraxane	6 (23%)
FOLFRINOX and Gem/Abraxane	4 (15%)
Time to progression (days)	120 (60–180)
Follow-up (days)	200 (111–289)

Number quoted is the number of patients in each category for categorical variables, and median value for numerical variables, while the number in brackets signifies percentage of all patients in the category and 25th to 75th percentile range respectively. Follow-up time is quoted for patients who did not progress as time to censoring

Each patient received 6 MRI scans with the same protocol, first at simulation (SIM), approximately 14–21 days before the treatment start, and then immediately prior to delivery of each of the 5 radiation fractions (F1-F5). Images were acquired on the MRIdian 0.35T MRI-Linac system using a balanced steady state free precession pulse sequence [16] using the following parameters: TR/TE = 3.33/1.43 ms, flip angle = 60 deg, 310 × 360 points, 144 slices, voxel size 1.5 × 1.5 × 3 mm. Gross Tumor Volume (GTV) was segmented by the radiation oncologist in the SIM scan, and the scans F1–F5 were subsequently overlaid and co-registered online to the SIM scan. Local manual rigid registration in 3 dimensions was performed in MRIdian treatment console software, directly prior to delivery of each radiation fraction to ensure the GTV is aligned to its position in the SIM scan. This rigid registration was applied to the GTV contour to propagate it from SIM to F1-F5 scans. Processing was performed in the Mirada software (Mirada RTx 1.8, Oxford, UK) by a qualified medical physicist.

### Image analysis and feature extraction

All images and Gross Tumor Volume (GTV) regions of interest (ROI) as segmented by the radiation oncologist (JF and SH) were saved in DICOM format and analyzed in MATLAB 2018b (MathWorks Inc., city, state) using custom written code. No spatial interpolation of the images was performed as uniform voxel sizes were used for all scans and patients, and number of intensity bins for texture and histogram feature quantification was fixed at 64.

Prior to image feature quantification, normalization was performed by dividing each image by the corresponding median signal value in the kidney, as practiced in MR radiomics [17] to account for technical intensity variation between imaging sessions. A Kidney ROI was drawn manually for each patient and in each scan in 3 equally spaced slices through the right kidney, as shown in Additional file 1: Fig. S1. Right kidney was chosen as an organ that can be reproducibly delineated in all the scans with no observed image artefacts sometimes seen in superficial areas or towards the peripheral slices. The analysis and rationale behind choosing this normalization approach is described in detail in the Results section. ROIs of the patients’ whole abdomen cross-section were automatically generated through image thresholding in 10 slices around the middle of GTV ROI but excluding the GTV. The change in median signal in the abdomen between the simulation and first fraction scans were compared to corresponding changes in the GTV, to quantify the contribution of systemic, technical changes in image intensity to GTV signal.

To avoid overfitting, given the small number of patients, feature extraction was initially confined to histogram features [18] (see Table 2 for feature list) extracted from the GTV to describe the tumor image characteristics. The ratio of each feature between last and first fraction (F5/F1) was quantified as a measure of imaging change after radiation (delta radiomics), and this metric was then analyzed.

**Table 2:**
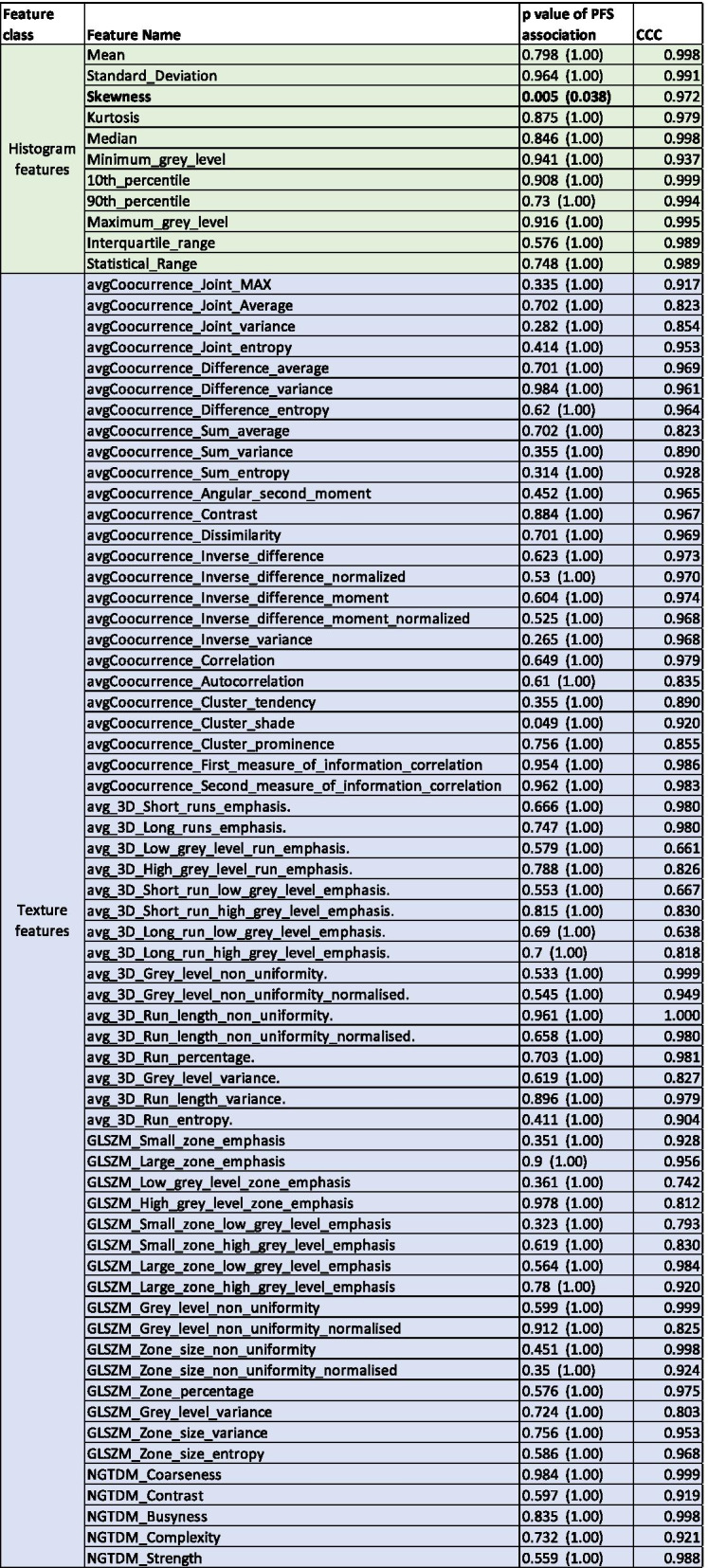
Feature quantification

Divided by histogram (green) and texture (blue), names of all quantified features (second column), corresponding *p* value of their association with Progression Free survival (column 3) pre and post (in brackets) multiplicity correction, and the Concordance Correlation Coefficient (CCC) values describing the spatial stability of each feature. P values pre-multiplicity correction are quoted to show the heterogeneity and dynamic range of associations

In the second part of analysis, N = 62 texture radiomic features were also extracted in 3D from the GTV, and delta radiomics ratios F5/F1 calculated. These features included: Grey Level Co-occurrence Matrix, Grey Level Run-Length Matrix, Grey Level Size Zone Matrix and Neighboring Gray Tone Difference Matrix features. The extraction protocol was consistent with the Image Biomarker Standardization Initiative [15] to ensure reproducibility of quantification.

Spatial stability of the features was tested by comparing the feature values in the SIM scan in ROIs created by radial erosion or expansion of the GTV by 1.5 mm in each slice, to simulate possible slight differences in GTV segmentation, and identify the least stable features to be avoided in further MRgRT radiomic analysis. Lin’s Concordance Correlation Coefficient (CCC) [19], commonly used for agreement quantification was then used to describe the robustness of the features to these small ROI changes.

### Statistical analysis

Comparison between imaging changes and progression free survival was performed in the n = 26 unresected patients. Sixteen progression events were observed, with median time to progression after radiotherapy of 4 months. Locally, a great majority of patients exhibited stable disease by RECIST, with 3 showing progressive disease, and 1 showing a partial response at latest follow-up CT scan.

The statistical analysis was performed using RStudio 1.2.5033 (RStudio Inc, MA, USA) interpreter. Univariate Cox proportional hazard regression model (*coxph* function, *survival* package in R) was used to assess the association between PFS and each feature ratio, separately in histogram and texture features, with Bonferroni-Holm correction applied for multiple comparisons. For features showing significant (*p* < 0.05) association, a conditional inference tree (*ctree* function, *partykit* package) was used to identify the high and low progression risk groups and quantify the optimal feature ratio threshold. The same approach was repeated for texture features.

## Results

Associations between the patient characteristics and progression-free surival were explored. Patients treated with Gem/Abraxane induction therapy showed significantly worse outcome (*p* = 0.005, HR = 9.45 (2.37–37.8) and HR = 4.63 (1.13–18.9) for Gem/Abraxane only and Gem/Abraxane after FOLFIRINOX, vs. FOLFIRINOX only). This is expected, as Gem/Abraxane tends to be prescribed at our institution to patients in worse overall condition who are less likely to tolerate FOLFIRINOX treatment. Resectability status at diagnosis showed no association with survival (*p* = 0.99).

Streamlined MATLAB code was written to enable integrated readout of the image and segmentation data and metadata, co-registration of the ROIs, normalization and quantification of image features in one pipeline for multiple patients at a time. This code, available at https://github.com/mrtomasz91/MRgRT_PDAC can be easily adapted and reused for further MRgRT image quantification studies.

**Fig. 1:**
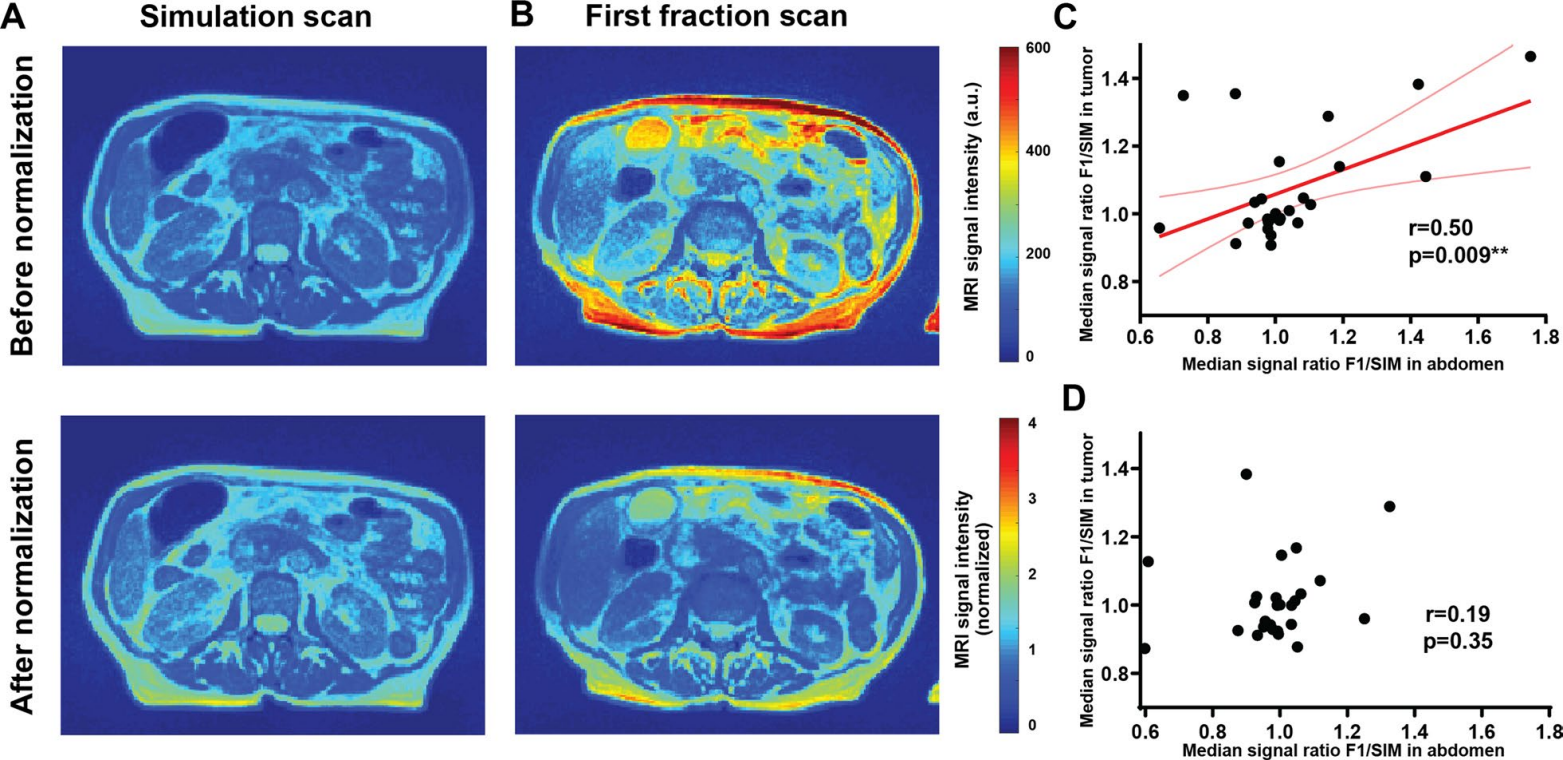
Linear image normalization removes global intensity variation. Images before (top row) and after (bottom row) normalization by division by median kidney signal are displayed. Strong global raw signal intensity changes between simulation (**A**) and first fraction (**B**) scan (when no treatment was administered) were observed, indicative of technical drift (no normalization, top row). This effect can be reduced by normalization (bottom row). The systematic correlation in image intensity changes observed between the tumor and rest of the abdomen (**C**), dominating the tumor intensity changes, is removed following image normalization (**D**)

Image pre-processing is often required for quantitative analysis of MRI data to account for the lack of internal normalization. The signal intensities in the SIM and F1 scans, when no treatment had yet been administered and therefore no biological trends were expected, were compared to identify technical signal changes unrelated to radiation response. As shown in a representative patient (Fig. 1A, top row), a clear overall change in the image scaling without normalization was apparent. Following this observation, the median signal intensity ratio changes between F1 and SIM scans were compared in the GTV vs. the rest of the abdomen, to quantify the extent to which the intensity changes represented global, technical scaling drifts, as opposed to tumor-specific changes. A significant positive correlation (Pearson r = 0.50, *p* = 0.009, Fig. 1C) was observed across the patient cohort between the signal ratios in the two regions, strongly suggesting that non-normalized GTV signal intensity is overwhelmingly affected by the technical image scaling unrelated to biological treatment response, highlighting the importance of pre-processing. Following normalization to median kidney signal, the image intensity change in a representative patient was reduced (Fig. 1A, bottom row), and the global correlation to overall image scaling was no longer present (Fig. 1D). Normalization by division was chosen based on the linear shape of the relationship in Fig. 1C.

Notably, no significant association was observed between pre-treatment (simulation scan) MRgRT image features and the PFS (*p* > 0.11). The prognostic power of the image histogram changes for PFS was then investigated. A significant association between PFS and the F5/F1 ratio for histogram skewness was observed based on Cox proportional hazard model (Hazard Ratio 2.75 (1.36–5.56), *p* = 0.038 post multiplicity correction). Other histogram features showed no association with disease progression (Table 1). The ROI volume, often strongly affecting feature quantification, showed no correlation with histogram skewness (*p* = 0.43), and no association with PFS (*p* = 0.40). Importantly the treatment variables -dose delivered and the number of days between the first and last radiation fraction were also not associated with PFS (*p* = 0.15 and *p* = 0.50 respectively).

**Fig. 2:**
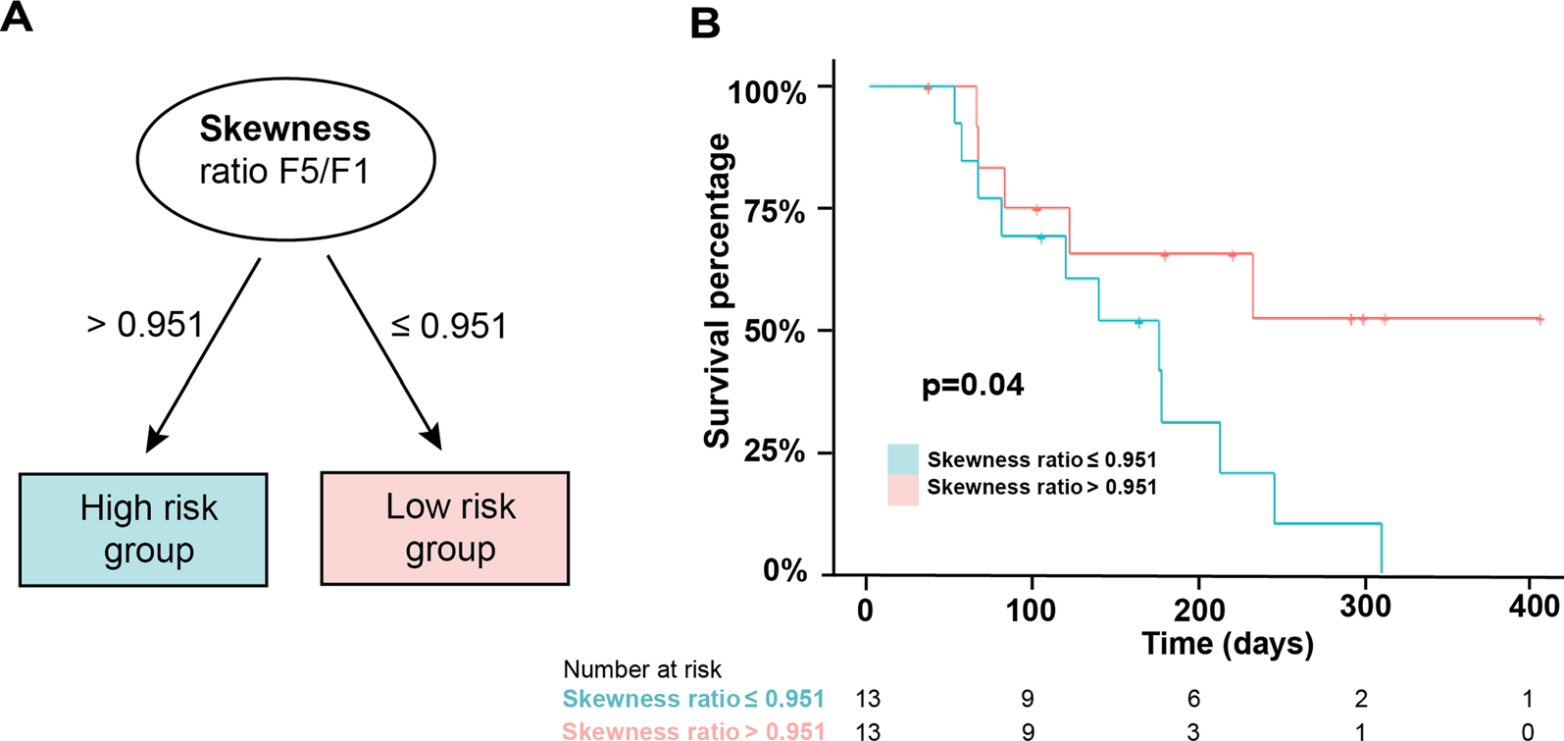
Histogram skewness change during treatment predicts progression free survival. Division of patients based on Skewness value ratio between 5th (F5) and first (F1) fraction image enables identification of high- and low progression risk groups (**A**). Kaplan–Maier analysis of progression free survival (PFS) within each group confirms a significant difference (**B**)

A conditional inference tree [20], a type of decision tree algorithm, was used to identify patient risk groups based on the Skewness change (Fig. 2), showing that the skewness of the F5/F1 ratio threshold can be used to differentiate patients with high risk of progression (Skewness Ratio > 0.95) from these with lower risk of progression (Skewness Ratio ≤ 0.95, Fig. 2A), with significantly different PFS between the groups (*p* = 0.041, see Fig. 2B for the Kaplan-Maier curves).

Association with PFS was also assessed for texture radiomic features. Contrary to the promising value of histogram quantification, despite over 60 features considered, borderline significant association (*p* = 0.049) was observed for one texture feature only (Cluster Shade), and only prior to multiplicity correction. The results are shown in Table 2. None of the histogram or texture features showed association with type of induction chemotherapy or resectability status.

**Fig. 3:**
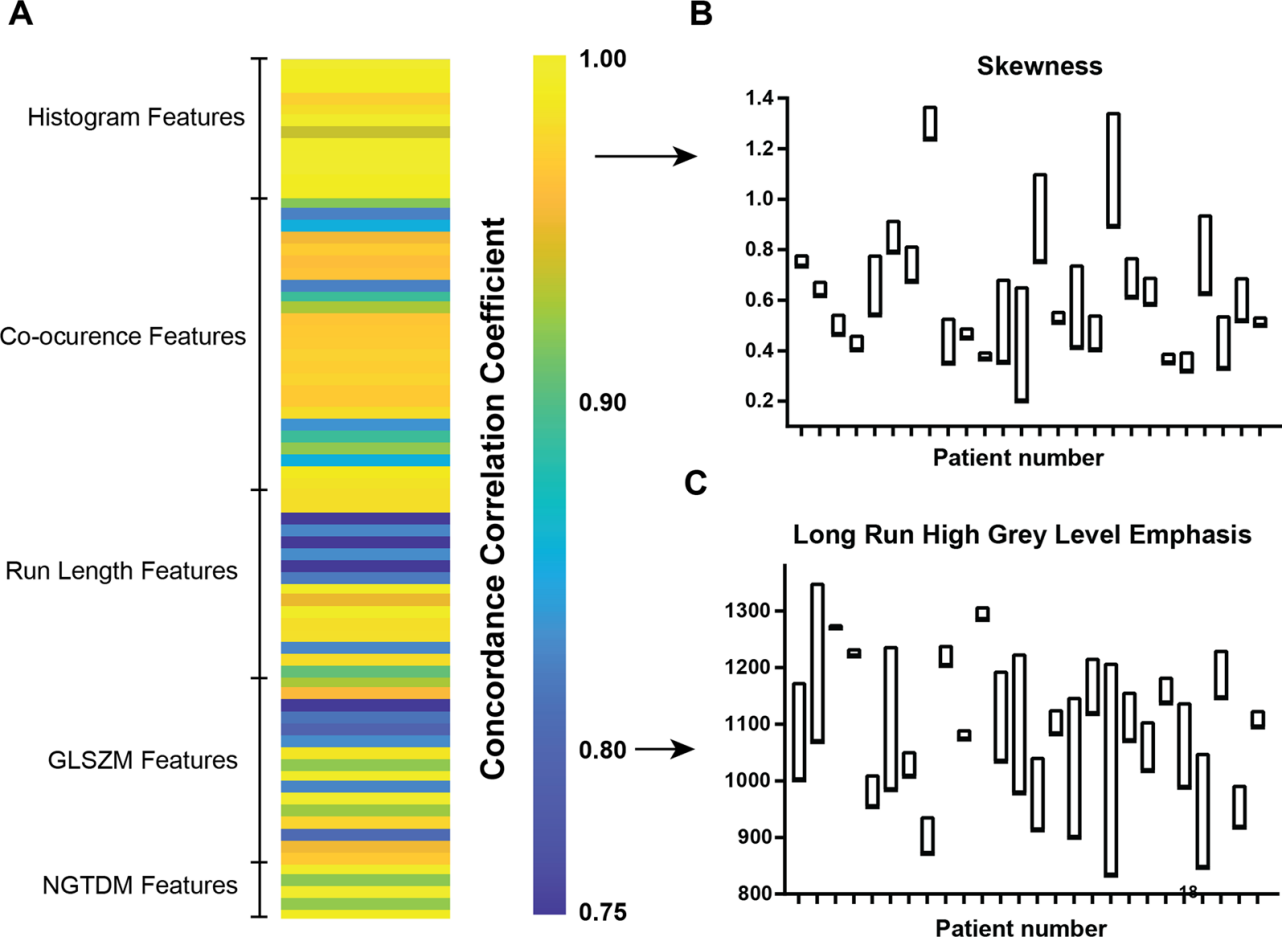
Robust analysis requires high spatial stability of features. Heat map (**A**) shows the distribution of Concordance Correlation Coefficient (CCC) for all quantified radiomic features, describing the robustness of the features to small changes in positions of the Region of Interest (ROI), calculated through translation of the ROI 1.5 mm in x and y. Results of this quantification for representative features with high CCC (Skewness, **B**) and lower CCC (**C**) are presented, showing a tighter distribution for repeated measurements for higher CCC. Bars denote distance from min to max value of the feature when ROI is shifted, with one bar for each patient

Analysis of spatial robustness of MRgRT radiomics was performed for both histogram and texture features through quantification of feature changes following small changes in GTV boundary position. The Concordance Correlation Coefficient (CCC) of the feature values after ± 1.5 mm GTV erosion and dilation, simulating minor differences in ROI drawing, was calculated for each feature as a measure of stability and the results are shown in Fig. 3. High heterogeneity and clear patterns by feature type are observed in spatial robustness (Fig. 3A). Good performance (CCC > 0.935) overall was measured for histogram features, a subset of co-occurrence matrix features related to inverse difference and a subset of Grey Level Size Zone Matrix (GLSZM) features related to non-uniformity. Importantly, skewness showed a good robustness compared to other features (CCC = 0.972, 62nd percentile). Serving as a visual example, the skewness values from shifted ROIs showed a tight distribution of each patient compared to the between-patient variability (Fig. 3B). On the other hand, poor spatial robustness, or high sensitivity of the feature to ROI shift as small as 1.5 mm, quantified in low CCC was noted for some features, e.g. grey level run emphasis features, as shown for High Grey Level Run Emphasis (Fig. 3C), for which the spread of the values for the perturbed ROIs is comparable to the dynamic range between patients.

**Fig. 4:**
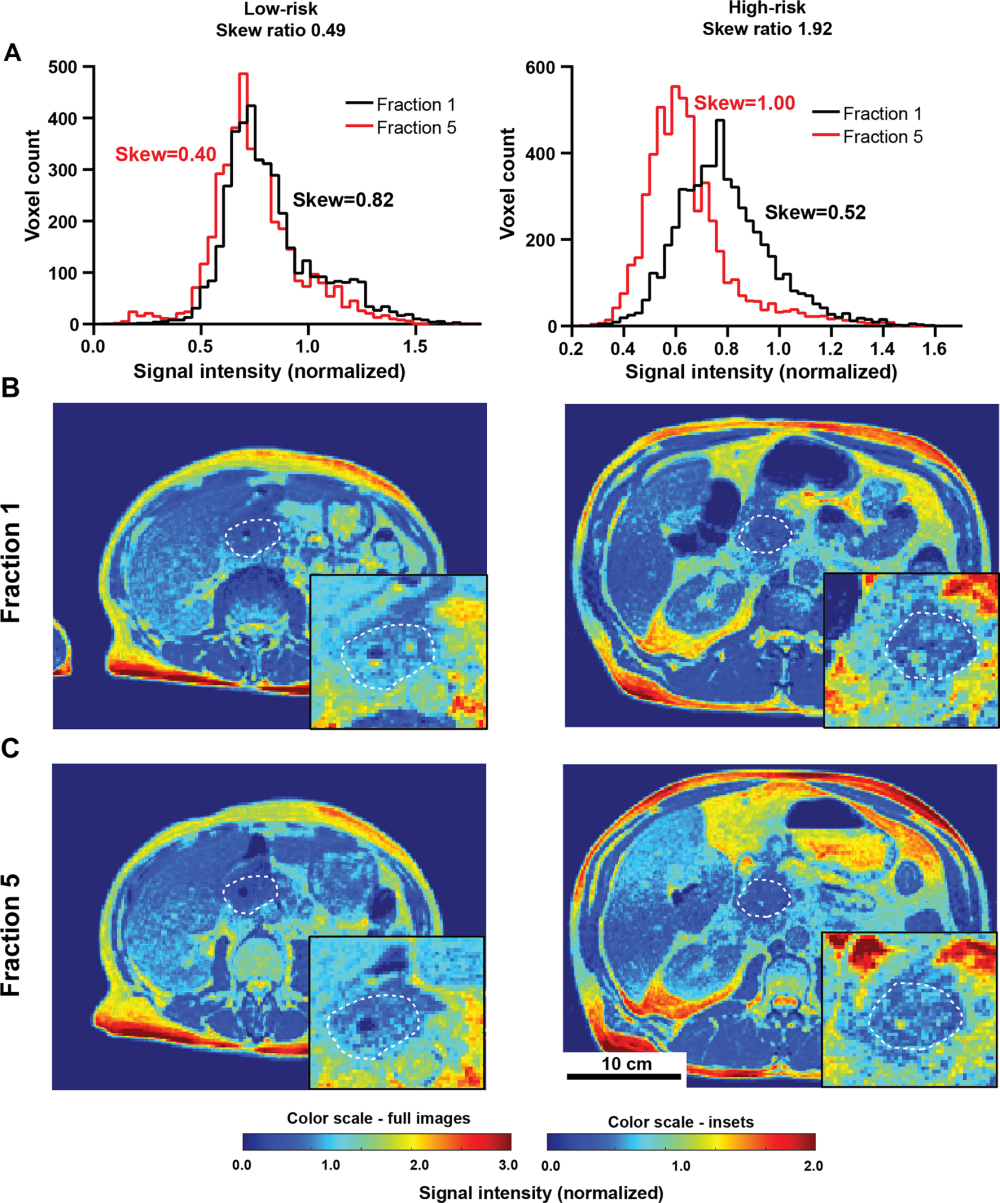
Skewness changes during treatment are not clearly visible in the images. Histograms of the GTV signal intensity distributions at first (black) and last (red) fraction are shown in (**A**) for representative low risk (left) and high risk (right) patients. Axial slices through the body for these patients at first and last (5th) fraction are shown in (**B**) and (**C**) respectively, outlining the tumor cross-section in dotted white line. Magnified tumor area is shown in insets.

Prior to treatment all but one tumor showed a mild positive (rightward) histogram skew (0.60 ± 0.06), with the group of patients more likely to progress (high-risk group) showing a further increase in right skewness during treatment (F5/F1 ratio = 1.52 ± 0.05), while the patients less likely to progress (low-risk group) showed a decrease in skewness to a more normal distribution (F5/F1 ratio 0.60 ± 0.01). The observed changes during treatment for representative low and high-risk patients and the corresponding image histograms are shown in Fig. 4, and while the histograms often shift considerably during treatment in both risk groups (Fig. 4A), these changes are difficult to discern visually in the images (Fig. 4B, C) highlighting the value of quantitative tools as presented in this paper.

## Discussion

Magnetic Resonance Guided Radiation Therapy constitutes one of the most impactful developments in recent radiation oncology technologies. Beyond the direct improvement in dose delivery precision and motion management, the abundance of real time imaging data opens significant opportunities for automated radiomic image quantification [8]. Our previous work highlights the promise of histogram change quantification for prediction of radiation response in preclinical models of PDAC [21] in MRI. The results of this study reinforce this view, showing that subtle yet quantifiable changes in the tumor imaging characteristics during the course of SBRT may be strongly associated with disease progression in pancreatic cancer, paving the way for image-driven dynamic dose adaptation.

We hypothesized that the dynamic information available from the MRI scans at each radiation fraction may provide particularly valuable insight, quantifying the early effects of irradiation on the tumor. Rather than considering the static image features at fixed timepoints, the analysis was therefore focused on parameter ratios at last vs. first fraction delivered. Quantifying the change in histogram parameters, widely used in MR imaging for heterogeneity analysis [18], a strong association of histogram skewness change with progression free survival was observed. A measure of the asymmetry of the voxel intensity distribution, the skewness was found to increase during treatment for patients with high progression risk, while in non-progressing low-risk patients the skewness was more likely to decrease. While highly statistically significant and with clear mathematical meaning, the changes are not visually apparent, highlighting the value of computational radiomic analysis for identification of hidden imaging patterns.

Beyond demonstrating the relationship with outcome, the study is the first to develop the protocol for MRgRT image analytics. We show that linear image normalization not only reduces signal variability between scans but is required to remove technical global signal drift and identify the tumor-specific signal intensity changes. As part of previously untested technical validation of MRgRT radiomics, we discuss the spatial robustness of the quantified radiomic features. The anatomical characteristics of PDAC lesions, notoriously challenging to segment reliably, highlight the relevance of this analysis. While some feature groups showed high technical variability following small changes in segmentation, suggesting poor reproducibility, the work revealed multiple robust features, promising for further radiomic studies. Conservative choice of small 1.5 mm ROI perturbation was designed to highlight the most unstable features to be avoided in future MRgRT radiomic analysis. Histogram features including the skewness were found to be highly spatially robust to these small changes. This is key for the clinical implementation of radiomic features into the clinical workflow. While this approach provides a robust automated framework for stability analysis, future work should also include direct measurements of inter- and intra-observer variability through repeated manual segmentation.

Progression free survival was chosen as the most appropriate and meaningful outcome metric, in line with the recent reports [5]. With a 60% event rate and follow-up as long as 13 months, the study was well-powered for identification of the observed progression patterns. Local control was not considered as the great majority of patients in this study (22/26) exhibited stable disease by RECIST, consistent with the low (10–20%) rate of local failure at 1 year in the literature.

Previous reports showed that radiomics may potentially be used to quantify image textures in MRgRT data, and relate them to outcome in pancreatic [12] and rectal cancers [10, 11] both in static and delta radiomic setting. Given the large number of features tested in small patient cohorts, as well as lack of image normalization, the results presented will require further validation. However, the rapidly increasing number of publications and conference presentations on the topic indicate significant interest in MRgRT radiomics. To minimize over-fitting in a small dataset, the analysis was limited to univariate signatures and focused firstly on histogram features. A larger study will be needed to verify the observed relationships, understand the link between radiomic features and treatment response, as well as to optimize the signatures through multivariate analysis. A higher-powered study may reveal further associations in the data not reported here, including relationships between pre-treatment imaging features and local control after irradiation, as reported in previous studies [12].

Given the increasing number of patients treated with this technology, a relatively simple pipeline required beyond standard of care to collect and analyze the data will enable quick accumulation of available data volume. This will soon allow for well-powered retrospective studies with 100 s of patients to be conducted. The uniformity of the acquisition settings between patients and imaging centers, especially on the 0.35T systems, will further accelerate the process, allowing for simpler multi-center collaborations, as already reported on a small scale [11]. Open access to the analytical pipeline developed in this study, including the necessary image processing and quantification code, may contribute to further simplification of the process.

Histogram features have been discussed in numerous MRI studies as descriptors of tumor heterogeneity [22, 23]. In particular the skewness of several MRI parameters, representing the asymmetry of voxel value distribution, has been reported to correlate with prognosis and outcome [18], especially in brain cancer [24]. Skewness changes during therapy were found to predict response [25, 26], as reported in this study, for the first time in MRgRT. However, understanding of precise biological underpinnings of these confirmed relationships poses a significant challenge [27]. Compared to some more established quantitative MRI metrics such as k^trans^ or ADC the Balanced Steady State Free Precession sequence signal intensity, used as a core sequence in 0.35T MRgRT, has a less well-defined biological meaning. Further work, including analysis of additional correlates may be required to provide a definite explanation. Insight may also be provided through histological comparison between the imaging and pancreatic resection specimens as part of a clinical trial underway at our institution.

The data presented above suggests that delta radiomics of Magnetic Resonance guided Radiotherapy imaging data shows significant promise for prediction of patient radiotherapy response, based on early changes in tumor morphology during radiation treatment, quantified in MR scans using histogram analysis. With further research, the findings may shed more light on the role of intratumor heterogeneity in radiotherapy response potentially increasing the reliability of predicting which patients can proceed to R0 resection, which can dramatically improve survival.

## Supplementary Information

**Additional file 1. Supplementary Figure 1:** Kidney Region of Interest. The regions of interest used for image normalization were drawn manually in three equally spaced slices of each scan. As shown in the example above, if the right kidney was present in slices 62-96, contours were drawn in slices representing ¼, ½ and ¾ of the way through the kidney volume, and voxels from these 3 slices used for the kidney signal intensity quantification.

## Data Availability

All materials are available on request, and analysis code can be downloaded from a public repository https://github.com/mrtomasz91/MRgRT_PDAC.
