# Supplementary material for: Delta radiomics analysis of Magnetic Resonance guided radiotherapy imaging data can enable treatment response prediction in pancreatic cancer
Source: Radiat Oncol. 2021 Dec 15;16:237. doi: 10.1186/s13014-021-01957-5 (PMC8672552; doi:10.1186/s13014-021-01957-5)
Supplement: Supplementary file 1 — Additional file 1. Supplementary Figure 1: Kidney Region of Interest. The regions of interest used for image normalization were drawn manually in three equally spaced slices of each scan. As shown in the example above, if the right kidney was present in slices 62-96, contours were drawn in slices representing ¼, ½ and ¾ of the way through the kidney volume, and voxels from these 3 slices used for the kidney signal intensity quantification. [file 13014_2021_1957_MOESM1_ESM.docx]

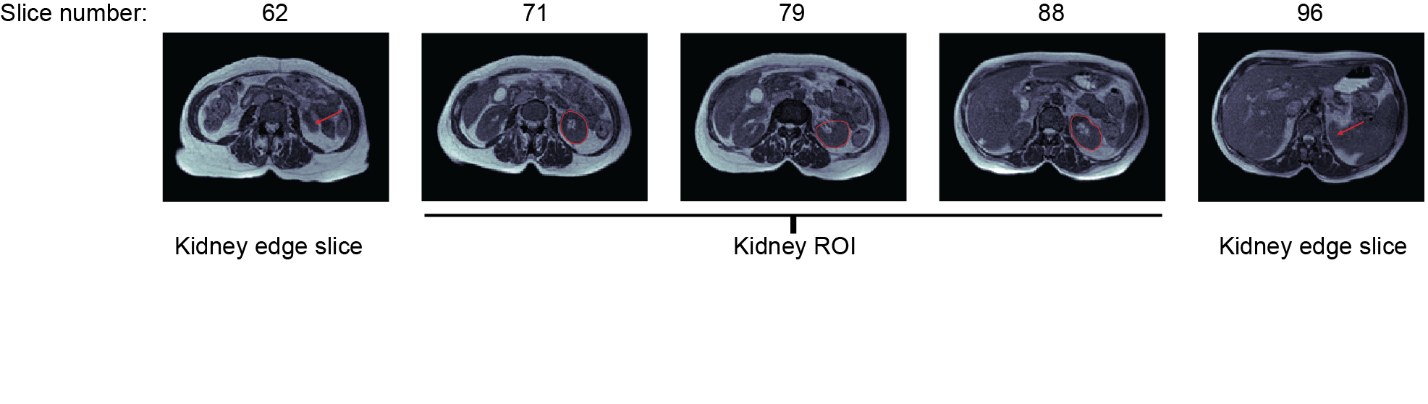


Supplementary Figure 1: **Kidney Region of Interest.** The regions of interest used for image normalization were drawn manually in three equally spaced slices of each scan. As shown in the example above, if the right kidney was present in slices 62-96, contours were drawn in slices representing ¼, ½ and ¾ of the way through the kidney volume, and voxels from these 3 slices used for the kidney signal intensity quantification.
